# 

**Published:** 1901-06-08

**Authors:** 


### NOTICE TO CORRESPONDENTS.

All MSS., Letters, Books for Review, and other matters intended for the Editor should be addressed The Editor, "The Hospital," The Hospital Building, 28 & 29 Southampton Street, Strand, W.C.

The Editor cannot undertake to return rejected MS., even when accompanied by stamped directed envelope.

**Notice to Advertisers and Subscribers.**

All Advertisements, Orders for Copies of The Hospital, and Business Communications should be addressed to **THE MANAGER (Not to the Editor),** The Hospital, 28 & 29 Southampton Street Strand, London, W.C.

The Cost of Subscription, post free, is as follows:—Per week, 2½d.; per quarter, 2s. 8d.; per half-year, 5s. 3d.; per annum, 10s. 6d. Foreign:—Per annum, 15s. 2d., payable, in all cases, in advance.

**NOTICE.—Vol. XXIX. of "The Hospital" (October 1900, to March, 1901), in handsome cloth binding are now on sale at the Office of this Journal, price, 7s. 6d. Cases for binding the Numbers contained in this Volume 1s. 6d. Index to Vol. XXIX., 2½d., post free.**

**The Monthly Part for June is now ready. Price 6d., by post 8d.**

### BOOKS RECEIVED.

J. B. Lippincott and Co.
"Diseases of the Nervous System." By H. Oppenheim, M.D. Translated by Edward E. Mayer, A.M., M.D.

Bale, Sons, and Danielsson.
"Outlines for Dissectors."

Simpkin, Marshall, Hamilton, Kent and Co.
"The Tea We Drink." By G. H. Skrine and George Browden, F.C.S.

J. M. Carr, Newcastle-on-Tyne.
"Health in the School." By Thomas Oliver, A.M., M.D., F.R.C.P.

A. Gallner Kamp and Co.
"Catalogue of Chemical and Other Apparatus."

Kegan Paul, Trench, Trubner, and Co.
"Arsenic." By Professor J. Alfred Wanklyn, M.R.C.S.

Philip Wellby.

"The Colour Cure." By A. Osborne Eaves.

Dawbarn and Ward.
"The Electrolytic Deposit of Sulphur from the Harrogate Sulphur Waters." By Francis William Smith, M.D., M.C., Aberdeen.

H. K. Lewis.
"Reports of the Medical, Surgical, and Pathological Registrars of the Middlesex Hospital for the Year 1898."

Adam and Charles Black.
"Jerusalem: a Practical Guide." By G. A. Reynolds-Ball, B.A., F.R.G.S.

Methuen and Co.
"Diseases of the Heart." By Edmund Henry Colbeck, B.A., M.D. Cantab., M.R.C.P., D.P.H.

The Scientific Press, Limited.
"Burdett's Official Nursing Directory, 1901." By Sir Henry Burdett, K.C.B. Fourth Year.

John Wright and Co.
"Golden Rules of Aural and Nasal Practice." By Philip R. W. de Santi, F.R.C.S.
"Golden Rules" Series.

P. Blakiston, Son and Co.
"3,500 Questions on Medical Subjects."

Frontier Press, Peshawar.
"Ague or Intermittent Fever." By Lieut-Col. Matthew D. O'Connell, M.D.

Horace Marshall and Son.
"Advice to 20th Century Business Juniors." By Phi, Pho, Chi.

A. Malonie, Paris.
"La Chaleur Radiante Lumineuse Agent Therapeutique." Par Le Docteur P. Guyénot.

Burns and Oates.
"Intemperance: Natural Remedies." By Professor Campbell, M.D.

Edward Arnold.
"The Physiological Action of Drugs." By M. S. Pembury, M.A., M.D., and C. D. F. Phillips, M.D., LL.D.

### SCRAPS AND GLEANINGS.

DURING the past year a sum of £1,000 has been expended by the Committee of the National Sanatorium for Consumption, Bournemouth, in improving the sanitary arrangements.

THE Sites Committee of the Devon and Cornwall Association for the Prevention of Consumption are busy collecting particulars as to available sites on which to build a sanatorium which it is hoped may accommodate not less than sixty patients.

FOUR exhibitions of the Roentgen Ray apparatus, which was recently given to the Essex and Colchester Hospital by Mrs. Hawkins, were given lately in the out-patients' hall of the hospital, the proceeds of which will go to the hospital's funds.

### THE STUDENT OF MEDICINE.

### APPOINTMENTS.

**G. F. Beacher,** L.R.C.P., L.R.C.S.Edin., appointed District Medical Officer of the Sheffield Union. **Vernon K. Blackburn,** L.R.C.P.Lond., M.R.C.S., has been appointed Assistant Resident Medical Officer to the Sheffield City Hospitals for the treatment of cases of infectious diseases. **A. G. Brinton,** M.R.C.S., L.R.C.P., appointed Senior House Surgeon to the Birmingham and Midland Eye Hospital. **R. C. Brown,** M.B., B.C.Camb., appointed Medical Officer of Health for the Diss Urban District. **M. A. Cooke,** M.R.C.S., L.R.C.P.Lond., appointed District Medical Officer of the Stroud Union. **P. H. Dunn,** L.R.C.P.Lond., M.R.C.S.Eng., appointed Medical Officer of Health for the Stevenage Urban District, **John Empson,** M.D., C.M., L.R.C.P.I., L.R.C.S.I., L.M., appointed Certifying Factory Surgeon for the Milborne

Port District of Somersetshire. **A. Emrys-Jones,** M.D., appointed Honorary Consulting Ophthalmic Surgeon to the Bolton Infirmary on his resignation of the post of Honorary Ophthalmic Surgeon. **A. E. H. C. Hallen,** M.D.Edin., appointed District Medical Officer of the Parishes of St. Giles and St. George, Bloomsbury. **A. Wellesley Harris,** M.R.C.S.Eng., D.P.H., appointed Medical Officer of Health for the Metropolitan Borough of Lewisham. **G. J. Line,** M.D.Dub., B.Ch., appointed District Medical Officer of the Woodstock Union. **John McOscar,** M.R.C.S., L.R.C.P., L.S.A., appointed Honorary Surgeon to the Victoria Cottage Hospital, Woking, Surrey. **C. Killick Millard,** M.D., D.Sc., appointed Medical Officer of Health and Public Analyst for the Borough of Leicester. **Herbert J. Paterson,** M.A., M.B., B.C.Cantab., F.R.C.S., appointed Assistant Surgeon to the London Temperance Hospital. **G. Melmoth Scott,** M.D., B.C.Cantab., appointed Resident Surgical Officer to the Birmingham and Midland Eye Hospital. **W. M. Smith,** M.B., appointed District Medical Officer of the Cheadle Union. **L. E. Stevenson,** M.B., B.C.Cantab., appointed District Medical Officer of the East Ward Union. **Henry J. Taylor,** M.R.C.S.Eng., L.R.C.P.Lond., appointed Honorary Ophthalmic Surgeon to the Bolton Infirmary and Dispensary. **A. S. Underhill,** M.D., T.C.D., D.P.H.Camb., appointed Medical Officer to the Workhouse of the West Bromwich Union. **W. Morley Willis,** F.R.C.S.Eng., appointed Honorary Assistant Surgeon to the General Hospital, Nottingham.

